# Performance evaluation of a hyphenated laser spectroscopy system with conventional methods for microplastic analysis

**DOI:** 10.1038/s41598-024-70501-8

**Published:** 2024-08-20

**Authors:** M. Vasudeva, U. K. Adarsh, Anish Kumar Warrier, Sajan D. George, V. K. Unnikrishnan

**Affiliations:** 1 Department of Atomic and Molecular Physics, Manipal Academy of Higher Education, Manipal, Karnataka 576104 India; 2 Department of Civil Engineering, Manipal Institute of Technology, Manipal Academy of Higher Education, Manipal, 576104 India; 3 Centre for Climate Studies, Manipal Academy of Higher Education, Manipal, 576104 India; 4 Centre for Applied Nanosciences, Department of Atomic and Molecular Physics, Manipal Academy of Higher Education, Manipal, 576104 India

**Keywords:** Environmental sciences, Natural hazards, Materials science, Optics and photonics

## Abstract

Microplastics are one of the concerning environmental pollutants because of their ubiquity. Their capability to adsorb other environmental pollutants increases the risk even further. Existing identification approaches for microplastic characterization for polymer class and their surface-adsorbed heavy metal detection require the utilization of multiple resources and expertise. The article discusses the applicability of a custom-made hyphenated Laser Induced Breakdown Spectroscopy (LIBS)—Raman spectroscopic system in characterizing microplastics by comparing the analytical performance with conventional methods such as Attenuated Total Reflectance- Fourier Transform Infrared (ATR-FTIR) spectroscopy, confocal Raman spectroscopy, and Scanning Electron Microscopy–Energy Dispersive X-ray Spectroscopy (SEM–EDS). Raman analysis identified polyethylene (PE), polypropylene (PP), and polyethylene terephthalate (PET) plastics, which is confirmed by confocal Raman and FTIR study of the same. LIBS study of microplastics detected heavy metals such as Al, Ni, Co, and Zn, along with Ca and Mg trace elements. The cross-examination with EDS validates these trace elements' presence on the microplastics' surface. The results of the reported LIBS-Raman analysis and its validity evaluated using conventional gold-standard methods show the applicability of the proposed methodology in characterizing microplastics from environmental resources with less or no sample preparation in short time.

## Introduction

Plastics are synthetic polymers consisting of long chains of monomers, which have become an essential part of our lives due to their durability^1^. However, the mismanagement of plastic waste is causing some concern because of its threat to humans and the environment^2–4^. This threat of solid plastic waste accumulation has multiplied in the past decade, and it is reflected in the abundance of microscopic plastic particles (microplastics) in the environment^5^. Microplastics refer to plastic particles having a dimension between 1 µm and 5 mm^6–8^. They are either produced in these dimensions for industrial applications, termed primary microplastics or are resulted from the degradation of larger plastics, termed secondary microplastics. Due to their increased specific surface area compared to the solid pieces, the adsorption of environmental pollutants like Persistent Organic Pollutants (POPs), bio-film, and heavy metals takes place^9^.

Heavy metals are metallic elements with a density^10^ greater than 5 g/cm^3^. They are considered hazardous environmental pollutants among these contaminants since they can form lipophilic ions/compounds when they form bonds with non-metallic molecules inside the living cell and can lead to cellular toxicity^11^. Anthropogenic activities like mining, usage of pesticides, and industrial wastewater introduce heavy metals into the environment^12^.

Due to the weathering of plastic fragments, the specific surface area (ratio of surface area to mass) increases with enhancement in surface roughness that aids in the adsorption of heavy metal contaminants onto the surface^13^. The adsorption rate of heavy metals among different classes of plastics itself varies and adds complexity further^14^. Some plastics also contain heavy metallic additives in their polymer matrix, which are added to attain the desired characteristics, and once waste plastics reach the environment, they can leach out from the polymer matrix^15^. Chen et al.^16^, with the help of an in-vitro digestion model, demonstrated the desorption of adsorbed heavy metal contaminants from the microplastics subjected to artificial gastric and intestinal juice, which is an example of the vector effect of microplastics in the translocation of heavy metals into the human body. Further, the potentiating effect of the adsorption and desorption of heavy metals with microplastics needs to be explored to assess their ill effects.

The current assessment approaches for microplastic contamination (characterization via polymer type and surface adsorbed heavy metals) are complex multistep processes. The existing methods for characterizing microplastics are visual (optical and electron microscopy^17–19^), thermochemical [Pyrolysis Gas Chromatography/Mass Spectrometry (Py-GC/MS), Thermogravimetric Analysis–Differential Scattering Calorimetry (TGA–DSC)]^20,21^, and spectroscopic (FTIR and Raman spectroscopy^22–25^) methods. For heavy metal identification, spectroscopic [EDS, Inductively Coupled Plasma–Optical Emission Spectroscopy (ICP-OES), X-ray Fluorescence (XRF), and LIBS^26–29^, and electrochemical methods have been used. Although the collection-extraction method of microplastics from environmental samples (predominantly water and sediment) is almost similar, the sample preprocessing steps required for different identification techniques are completely different^30^.

The necessity of applying multiple analytical methods for microplastics and their surface-adsorbed heavy metal detection hinders rapid characterization and demands the use of several harsh chemicals for multistep sample preparation and analysis as well. These methods also demand the usage of multiple resources. To address this barrier of the multistep analysis process, the integration of complementary techniques under a single analytical system that shares a similar instrumentation design has proven to be practical for different analytical problems^31^. One such system is a bimodal LIBS-Raman spectroscopy, which combines elemental and molecular identification^32^. The combination of LIBS and Raman spectroscopy under a single system has several advantages over other techniques, such as cost-effectiveness, minimal sample preprocessing, and rapid analysis^33^. The first-hand results on the microplastic-heavy metal detection using the LIBS-Raman system can be found in our earlier publication^30^.

In this article, we examine, in detail, the potential of the LIBS-Raman spectroscopy system in characterizing microplastics from environmental samples by comparing the results with conventional identification techniques and validating the characterization performance. We utilize Raman spectroscopy to characterize microplastics and LIBS to diagnose surface-adsorbed heavy metals. The system's ability to completely characterize microplastics using the proposed system is demonstrated by comparing the results with existing gold standards like ATR-FTIR, confocal Raman spectroscopy, and SEM–EDS. The developed methodology requires a simple sample extraction and pre-treatment steps, which helps the rapid analysis.

In comparison with our earlier publication^32^, this article discusses the validation of a LIBS-Raman spectroscopic system in identifying the polymer class as well as the adsorbed heavy metal contaminants on microplastics in the size range of 1–5 mm with conventional methods. A total of six microplastic samples collected from an estuary were characterized by the LIBS-Raman spectroscopic system and the commercial systems, namely, ATR-FTIR and SEM coupled with EDS, and the results were evaluated to illustrate the competence of the system over traditional techniques requiring multiple systems for complete analysis. The comparison of results obtained by the lab-built LIBS-Raman system is at par with conventional methods.

## Methodology

### Collection and extraction of microplastics

For the current study, the microplastic samples were collected from an estuary on the west coast of India, formed by the River Netravathi in Mangalore, Karnataka, India. From the sampling site, 100 L of water was collected using a 10 L stainless-steel bucket and subjected to sieving with the help of a stack of stainless-steel sieves with pore sizes 5 mm and 1 mm. Before collecting the River water, all equipment was rinsed with distilled water, followed by river water. Then, water was collected using the stainless-steel bucket and poured into the stack of sieves. The macro debris (> 5 mm) that remained on the top of the sieve (with 5 mm pore size) was discarded, and the residues collected in the sieve (with 1 mm pore size) were transferred to a stainless-steel container with the help of distilled water, The sample was subjected to preprocessing at the laboratory using the extraction protocol by Masura et al.^34^ and Amrutha et al.^35^.

In brief, the samples were subjected to wet sieving with stacked stainless-steel sieves with 5 mm and 1 mm pore sizes. The residue was transferred to a glass beaker and kept in a hot air oven for a duration of 24 h at 50 °C. Then, the samples were subjected to Wet Peroxide Oxidation (WPO) to digest the organic matter with the help of 20 mL of 30% v/v hydrogen peroxide (H_2_O_2_). Then, 20 mL of 0.05 M ferrous solution was added to the beaker and heated using a hot plate. Hydrogen peroxide was added drop by drop till all the organic material was dissolved. This procedure is necessary to remove the organic contaminants residing on the microplastics' surface, which will aid in extracting microplastics from the sample by adopting the density separation method. Although, the WPO with hydrogen peroxide will change the valency of the heavy metal contaminants when the concentration of H_2_O_2_ used is effectively high^36^, in our study, we used 20 mL of 30% H_2_O_2_ solution, which is only sufficient to digest the organic contaminants and has minimal effect on the change in the valence state of the metal ions leading to the desorption. Also, from our early reports, it is clear that the WPO process with the specified concentration of hydrogen peroxide dissolves only the organic surface contaminants and does not induce any chemical or structural changes to the microplastics within 24 h, which is evident from Raman spectral analysis of microplastics with and without WPO^30^. For the selection of suitable salt for extracting the microplastics, a literature survey was conducted, and the results are as follows. Several studies have been conducted with different salt solutions, including NaCl, NaBr, and ZnCl_2,_ on the aspect of microplastic recovery from different environmental samples like sediment, water, soil, etc. The recovery rate observed by Radford et al.^37^ in extracting microplastics from soil samples with NaCl solution (1.2 g/cm^3^ density) was around 60%, whereas Vermeiren et al.^38^ achieved > 90% recovery using ZnCl_2_ solution (1.7 g/cm^3^ density)^37–39^. Hence, ZnCl_2_ was chosen for density separation, which has a recovery rate of > 90%. In order to increase the density of the aqueous solution, ZnCl_2_ solution was added to the beaker (concentration 933.3 g/L). Then, the mixture was transferred to a density separator and kept aside to allow the denser organic materials to settle down. Since the density of polymers, like 1.3 g/cm^3^ for PVC and 1.45 g/cm^3^ for PET, is lesser than the density of the ZnCl_2_ solution (1.7 g/cm^3^) used in our study, the recovery rate will be maximum. The presence of small amounts of heavy metal contaminants does not significantly change the density of these microplastics; hence, to the best of our understanding, their recovery rate will not be affected. Following this, the organic matter that got settled down was drained off, and the microplastics floating in the supernatant were sieved once again with 5 mm and 1 mm sieve, transferred to a glass container, and dried.

A total of six microplastics were extracted and were named MPS1, MPS2, MPS3, MPS4, MPS5, and MPS6. The characterization of the extracted samples was done based on their morphology, polymer class, and adsorbed heavy metals.

### Visual identification

The microplastic samples were transferred into a watch glass and visualized under the Nikon Eclipse Ni microscope (40× and 100× magnification) to analyze their morphology. The brightfield images were captured with resolutions of 2.12 μm and 0.92 μm with 4× and 10× objective lenses. Then, they were categorized into fragments, films, and fibers based on shape and size^34^.

### Identification of polymer class

A locally developed LIBS-Raman spectroscopic system was used to characterize the microplastics, and a detailed description of the system is discussed in our previous article^32^. In brief, a frequency-doubled, Q-switched Nd:YAG laser (Quantel Q-smart 450) was used as an excitation source. The laser operates at 532 nm with a 6 ns pulse width and 10 Hz repetition rate. The LIBS or Raman signals are collected using a back-collection optical configuration.

The Czerny–Turner spectrograph (Kymera 328i, Andor) with multiple grating options (600 lines/mm, 1200 lines/mm blazed at 500 nm, and a holographic grating 2400 lines/mm blazed at 300 nm) is used for Raman analysis. In the current study, 600 lines/mm grating with ~ 130 nm bandpass is used for the spectroscopic analysis. The spectra were recorded using a CCD detector (iDus -420, Andor) operating with the synchronized trigger from the laser.

The LIBS spectral analysis was carried out by coupling the atomic emission signals from the sample to the Echelle spectrograph (Mechelle ME5000, Andor) with an ICCD detector (iStar, Andor) using an optical fiber cable. The spectrograph has a spectral coverage of around 775 nm in a single scan with a high resolution of 0.01 nm. The ICCD was synchronized with the laser source via the Digital Storage Oscilloscope (DSO) for gate monitoring. The LIBS spectra were recorded in single-shot mode, with optimized gate width and gate delay as 10 μs and 700 ns, respectively.

#### Microplastics analysis using Raman spectroscopy

The polymer class of the collected microplastics was identified using Raman spectroscopy with the help of the LIBS-Raman system. The microplastic samples were sandwiched between two glass slides as prescribed in a study^30^. The extended part of the microplastics out of the slides was introduced at the focal point of the focusing lens. The energy of the laser beam was reduced to 0.8 mJ using the energy regulator, and with external triggering from the laser, the spectra were recorded with an exposure time of 5 s in the spectral range 278–3760 cm^−1^ with a resolution of 2.76 cm^−1^. The collected Raman spectra were baselined using the asymmetric least square method with OriginPro software. The polymer class of each microplastic was identified by comparing the Raman spectra of standard polymer samples and with the literature^40–45^.

Further, the samples were also analyzed under a commercial Raman microscope (XpolRA PLUS, HORIBA Scientific) with a 532 nm laser source, 20× objective lens, and 1800 lines/mm grating to compare the lab-based system's ability. The Raman spectra were acquired with 2 s acquisition time. The obtained Raman spectra were smoothened using the Savitzky–Golay method with a window size of 10 points and baselined using the asymmetric least square method with OriginPro (OriginPro 2022 (9.9) SR1, OriginLab, https://www.originlab.com/index.aspx?go=Products/Origin/2022&pid=4418) software.

#### Microplastics analysis using ATR-FTIR

For the cross-verification of polymer class identification, we took FTIR spectra of analyzed MPs using ATR-FTIR (Spectrum two, PerkinElmer) apparatus. The spectra were collected in the wavenumber region 600–4000 cm^−1^ with a spectral resolution of 8 cm^−1^. Six MP samples were analyzed. The obtained spectra were baselined with OriginPro (OriginPro 2022 (9.9) SR1, OriginLab, https://www.originlab.com/index.aspx?go=Products/Origin/2022&pid=4418) software. The spectral assignments were manually done with the help of literature^46–49^.

### Adsorbed heavy metal identification

#### LIBS analysis of microplastics

The microplastic samples were subjected to LIBS analysis to identify the surface-adsorbed heavy metals. The laser energy was increased to 4 mJ, and the gate delay was set to 700 ns with a gate width of 10 μs. The LIBS spectra acquired were analyzed for adsorbed contaminants referring to the NIST (National Institute of Standards and Technology) atomic spectra database.

#### SEM–EDS analysis of microplastics

The cross-verification of the LIBS results on adsorbed heavy metal contaminants was performed along with the determination of the extent of degradation of microplastics, using the gold-standard methods-Scanning Electron Microscopy (SEM) and Energy Dispersive X-ray Spectroscopy (EDS). The microplastics were subjected to sputter coating with gold to develop a thin film of gold on the surface of the microplastics, and the SEM analysis was carried out by fixing the samples onto a stainless steel substrate and analyzed using Hitachi S-3400, operating at 15 kV. The elemental mapping of the microplastics with EDS characterization was carried out using a ThermoNORAN NSS detecting system.

## Results and discussions

### Visual identification and characterization of microplastics

**Figure 1:**
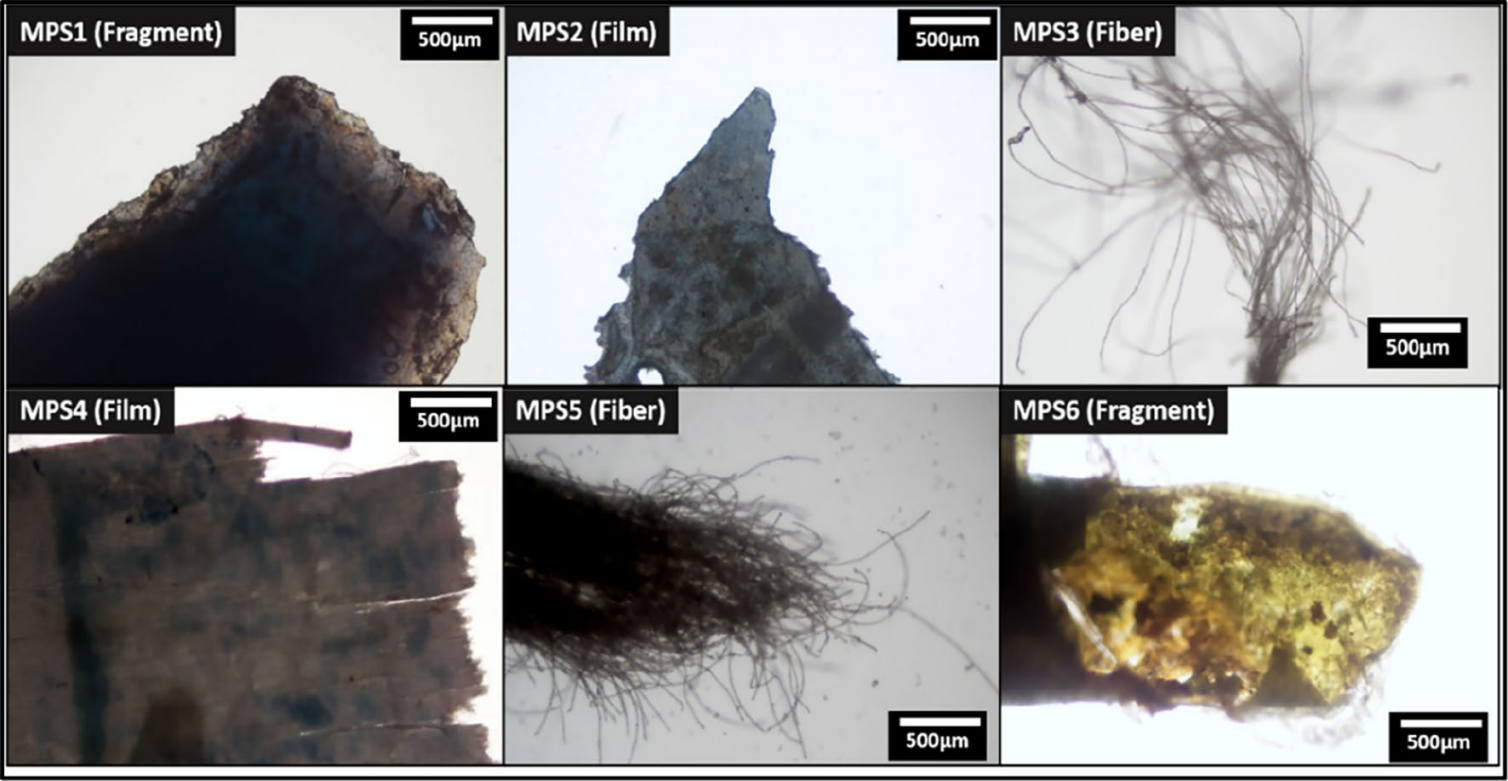
Brightfield images of the collected microplastics with naming.

The brightfield images of the six microplastics are shown in Fig. 1. Out of six microplastics, two are identified as fibers, two as fragments, and the remaining two as films. The sources of fibrous microplastics and films can be attributed to the discharge of fibers from synthetic cloths and single-use carry bags^35^. Based on the microplastics' morphological characteristics, all are identified as secondary microplastics because of their roughed and degraded surfaces^50^.

### SEM analysis of microplastics

**Figure 2:**
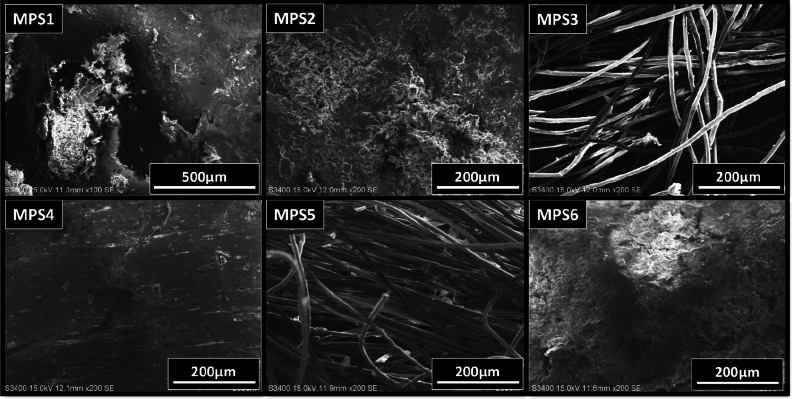
SEM images of the six microplastics.

The SEM images of the microplastics are shown in Fig. 2. After conducting all other characterizations mentioned in the methodology section, the SEM study was carried out as the conductive coating of gold is necessary for SEM characterization. The SEM images of the six microplastics revealed their highly degraded surface. Sample MPS1 had an uneven, roughened surface with craters and pits caused by weathering^51^. Sample MPS2 had a flaky surface, possibly caused by aberrant degradation^50^. Sample MPS3 was fibrous, and some microfractures were observed with smooth surfaces. Sample MPS4 had the least amount of surface degradation among the collected microplastics, with only a few minor fractures and cracks. Sample MPS5 was also fibrous, with a comparatively rougher surface than MPS3, and some flakes were embedded between the fibers. Sample MPS6 had a high degree of degradation, with protrusions, cracks, and a flaky surface.

### Polymer classification

#### Raman spectroscopic characterization

**Figure 3:**
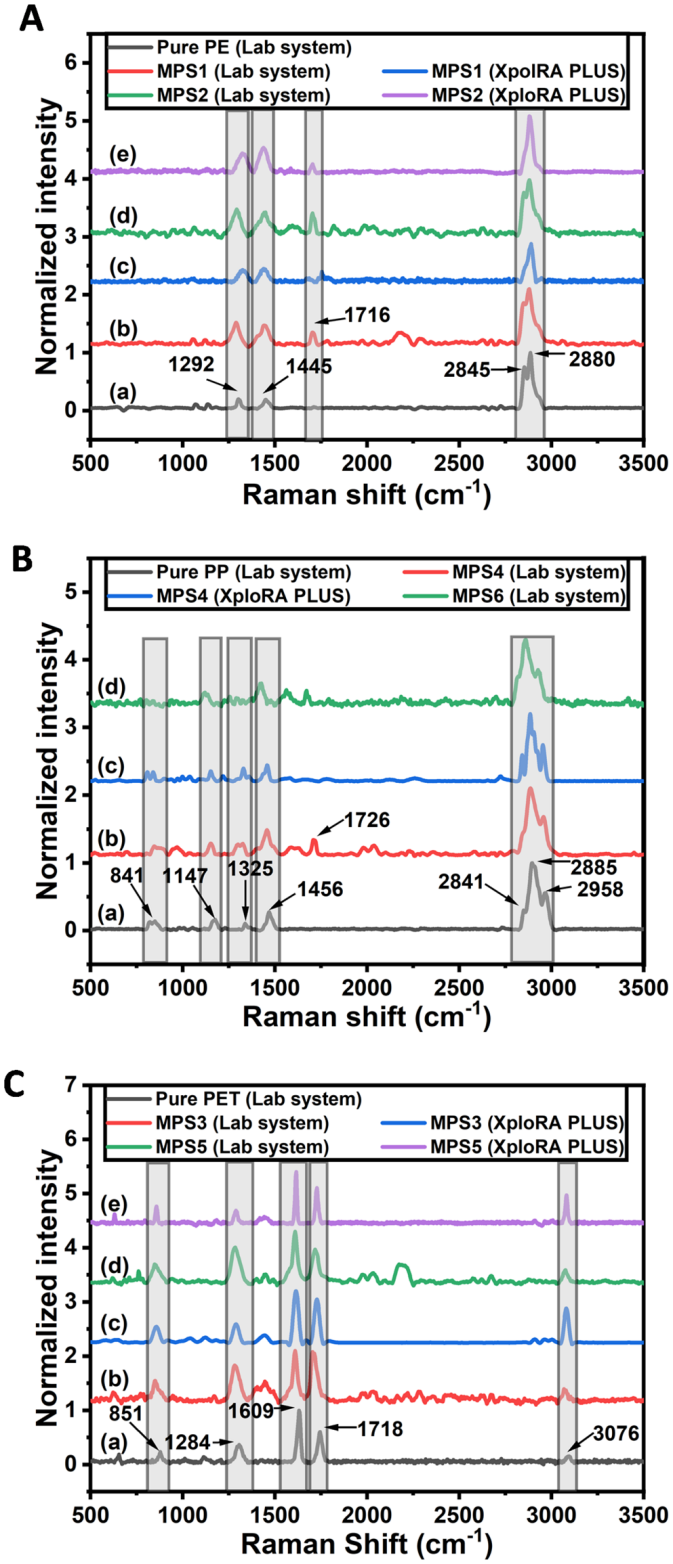
Raman spectra of the microplastics acquired using lab-built and commercial confocal Raman setup with peak assignments (**A**) Raman spectra of PE microplastics, (**B**) Raman spectra of PP microplastics, (**C**) Raman spectra of PET microplastics.

The Raman spectral comparison of extracted microplastics is shown in Fig. 3. Sample MPS1 and MPS2 were identified as polyethylene with two eminent peaks at 1292 cm^−1^ and 1445 cm^−1^, which were designated to CH_2_ twisting CH_2_ symmetric deformation. Two more peaks in the region 2880 cm^−1^ and 2845 cm^−1^ corresponding to CH_2_ asymmetric and symmetric stretching vibration were also observed. Apart from the characteristic peaks, a peak at 1716 cm^−1^ was observed in the Raman spectra of sample MPS1. It was assigned to C=O stretching vibration, which might be due to weathering-induced oxidation^30^. Sample MPS3 and MPS5 were identified as polyethylene terephthalate with characteristic peaks at 1609 cm^−1^ and 1718 cm^−1^ which were due to the ring mode vibration and C=O stretching vibration. Peaks corresponding to C–H stretching and C(O)–O stretching vibrations were observed at 3076 cm^−1^ and 1284 cm^−1^, respectively. The peak at 851 cm^−1^ corresponds to A_g_ mode ring C–C stretching vibration or C–H out-of-plane bending vibration. Other two samples, namely MPS4 and MPS6, were found to be polypropylene as the Raman spectra of those two microplastics exhibited peaks at 2958 cm^−1^, 2885 cm^−1^, and 2841 cm^−1^, which were due to CH_3_ asymmetric, CH_3_ symmetric and CH_2_ symmetric stretching vibrations respectively. Apart from those three peaks, three more peaks at 1456 cm^−1^, 1325 cm^−1^, and 1147 cm^−1^ were also observed, which were assigned to CH_3_ asymmetric bending, C–H symmetric bending, and C–C stretching vibrations.

Cross-verifying the polymer class of the microplastic samples was carried out under a commercial Raman microscope (XpolRA PLUS, HORIBA Scientific) with a 20× objective lens. The Raman spectra were acquired using LabSpec 6 software with 2 s acquisition time and 200 µm slit width. A comparison of the normalized spectra obtained using the lab-based and commercial systems is shown in Fig. 3. The spectral signatures observed by both systems are similar, and the spectral assignments are tabulated in Table 1. Thus, we can conclude that our system can efficiently distinguish between different classes of plastics.

**Table 1:** Spectral assignment corresponding to Raman spectra of microplastics.

Polymer class	Raman shift (cm^−1^)	Assignment	References
Polyethylene (PE)	1292	CH_2_ twisting vibration	^40,41^
	1445	CH_2_ symmetric deformation	
	1716	C=O stretching vibration	
	2845	CH_2_ symmetric stretching	
	2880	CH_2_ asymmetric stretching	
Polypropylene (PP)	1456	CH_3_ asymmetric bending	^42,43^
	1726	C=O stretching vibration	
	2841	CH_2_ symmetric stretching	
	2885	CH_3_ symmetric stretching	
	2958	CH_3_ asymmetric stretching	
Polyethylene terephthalate (PET)	851	A_g_ mode ring C–C stretching vibration or C–H out of plane bending vibration	^44,45^
	1284	C(O)–O stretching vibration	
	1718	C=O stretching vibration	
	3076	C–H stretching vibration	

#### ATR-FTIR analysis

**Figure 4:**
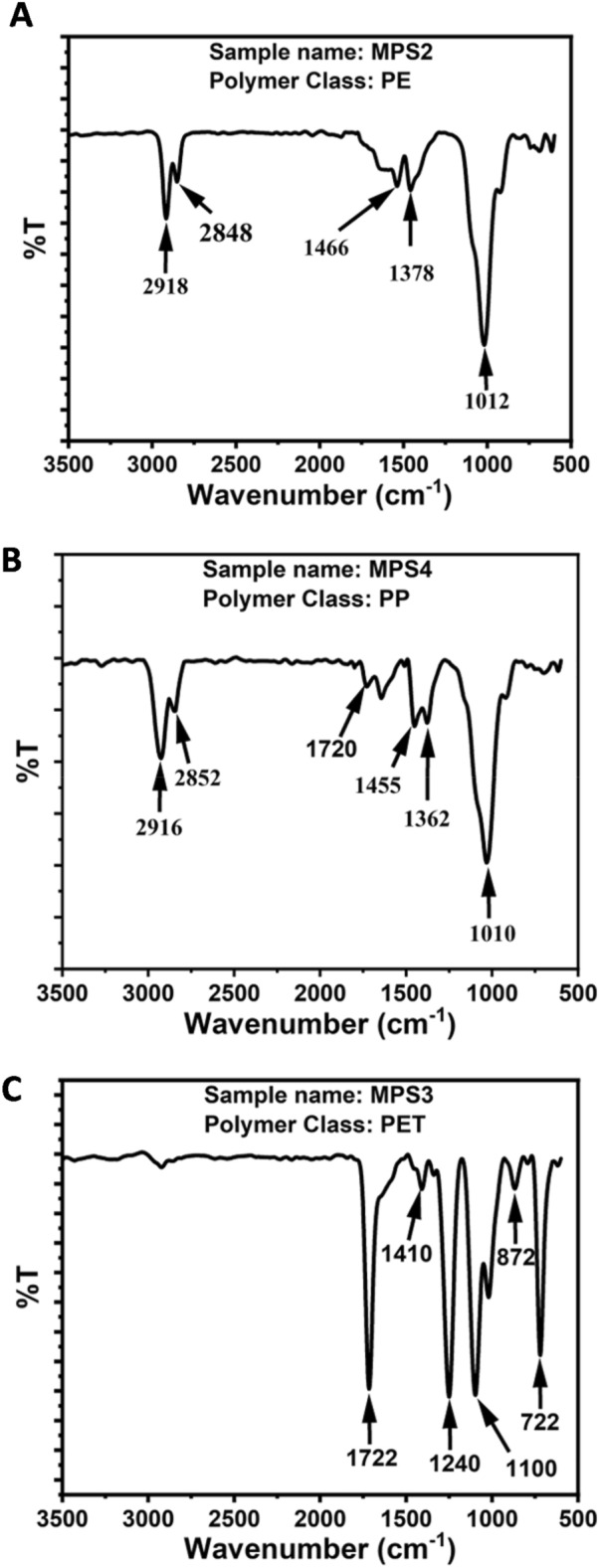
FTIR spectra of microplastics with characteristic absorption peaks observed: (**A**) MSP2 observed as PE, (**B**) MSP4 observed as PP, and (**C**) MSP3 observed as PET.

The ATR-FTIR analysis of the collected microplastics using the commercial system was carried out to further cross-verify the results obtained from the system based on Raman spectroscopy as they are complementary to each other in terms of molecular structure-based analysis. The FTIR spectra were collected by placing the microplastics onto the ATR crystal made up of diamond, and the spectra were analyzed in the wavenumber range of 600–4000 cm^−1^. The FTIR spectra of three samples, MPS2, MPS3, and MPS5, are shown in Fig. 4. The spectral assignments corresponding to the microplastic samples are tabulated in Table 2.

**Table 2:** Spectral assignment corresponding to FTIR spectra of microplastics.

Polymer class	Raman shift (cm^−1^)	Assignment	References
Polyethylene (PE)	1378	CH_2_ waging deformation	^46^
	1466	CH_2_ scissoring deformation	
	2848	CH_2_ symmetric stretching	
	2918	CH_2_ asymmetric stretching	
Polypropylene (PP)	1370	CH_3_ asymmetric bending	^47^
	1455	CH_2_ symmetric bending	
	1720	C=O stretching vibration	
	2852	CH_2_ symmetric stretching	
	2916	CH_2_ asymmetric stretching	
Polyethylene terephthalate (PET)	722	C–C stretching vibration	^48,49^
	1102; 1244	C(O)–O stretching vibration	
	1406	CH_2_ bending vibration	
	1720	C=O stretching vibration	

The noise level of the FTIR spectrum of MPS1 was very high, and thus, we were not able to identify the polymer class. The FTIR spectrum of MPS2 resembled the FTIR spectrum of polyethylene. The peak in the region 1378 cm^−1^ was due to the wagging deformation of the CH_2_. The peak in the region 1466 cm^−1^ was due to the CH_2_ scissoring. The peaks at 2848 cm^−1^ and 2918 cm^−1^ were assigned to the symmetric and asymmetric stretching of CH_2_. Interestingly, the additional peak at 1018 cm^−1^ was observed, which might be due to the presence of ester groups due to the photo oxidation of MPs. The FTIR spectra of MPS4 and MPS6 resembled the FTIR spectrum of polypropylene. The peak in the region 1370 cm^−1^ was assigned to the symmetric bending of CH_3_. The peak at 1450-1456 cm^−1^ was due to the symmetric bending of CH_2_. The peaks in the region 2830–2852 and 2916–2944 cm^−1^ were assigned to the symmetric and asymmetric stretching of the CH_2_ group. Two additional peaks between 996-1010 cm^−1^ and 1700–1720 cm^−1^ were observed, which might be due to the presence of ester and carboxyl groups due to the photo oxidation of MPs. The FTIR spectra of MPS3 and MPS5 resembled the FTIR spectrum of polyethylene terephthalate. The peaks in the spectral range 722-732 cm^−1^ and 872 cm^−1^ were due to the out-of-plane wagging and bending of aromatic C–H. The peaks in the region 1100-1102 cm^−1^ and 1224-1240 cm^−1^ were due to the C–O–C and C–C–O stretching vibrations. The peak in the region 1406–1418 cm^−1^ was assigned to the vibration of the phenyl ring. The peak at 1722 cm^−1^ was assigned to the C=O stretching of the carboxyl group. Two peaks corresponding to in-plane and out-of-plane vibration of the benzene group were identified at 1017 cm^−1^ and 868 cm^−1^, respectively. Interestingly, the additional peak at 1022–1024 cm^−1^ was observed, which might be due to the presence of ester groups due to the photo oxidation of MPs.

### Adsorbed heavy metal identification

#### LIBS analysis of microplastics

**Figure 5:**
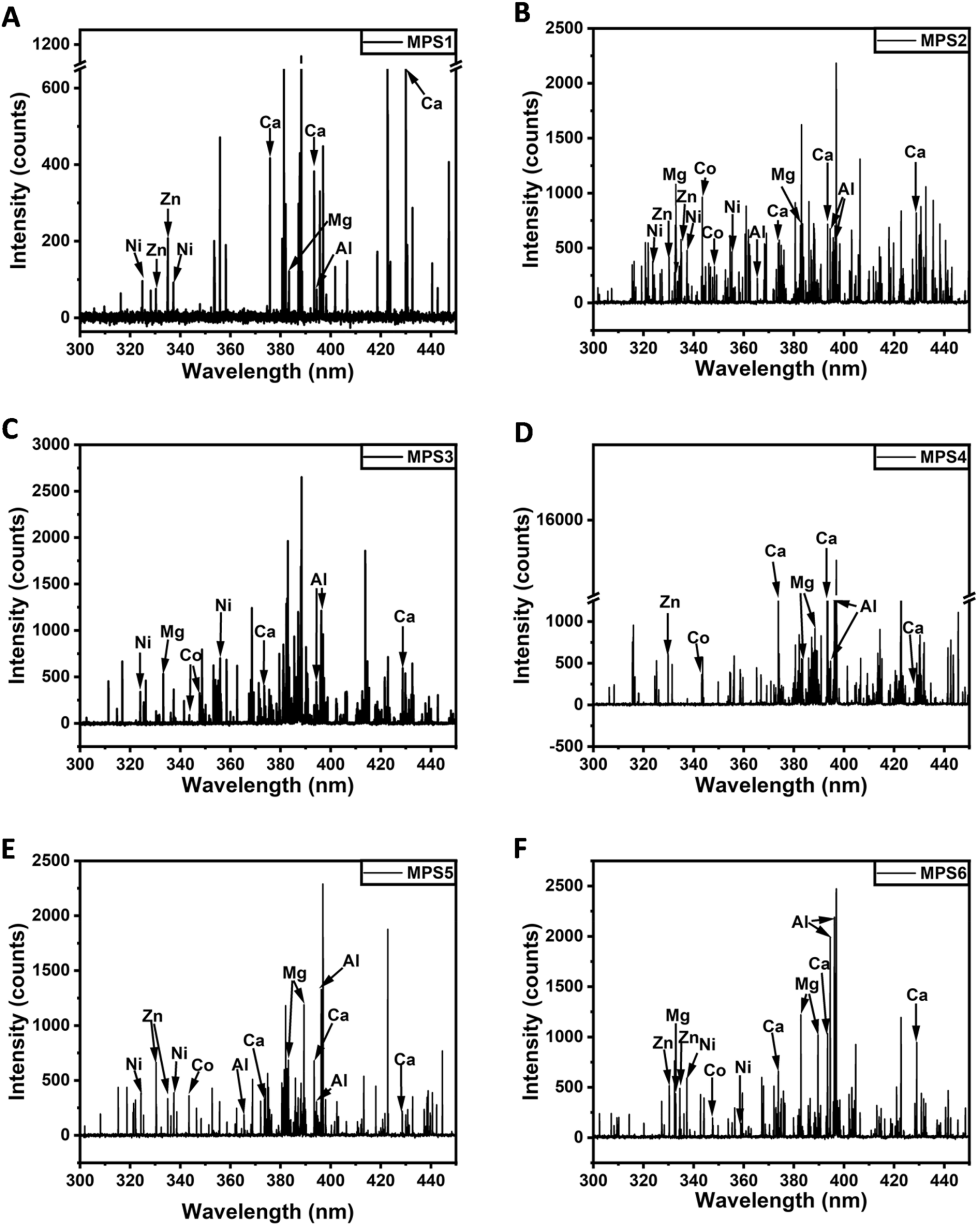
LIBS spectra of the microplastics along with the elemental assignment: (**A**) MPS1, (**B**) MPS2, (**C**) MPS3, (**D**) MPS4, (**E**) MPS5, (**F**) MPS6.

The LIBS spectra were recorded in the 200–900 nm spectral range with 10 ms exposure time and analyzed in the 300–450 nm spectral range to identify the heavy metals adsorbed onto the MPs. The spectral range was selected based on the presence of intense emission lines from heavy metals that were adsorbed onto MP's surface. The spectral assignments were done by comparing the emission lines with the NIST database. LIBS spectra of all six samples are shown in Fig. 5. Elements detected from all six samples with corresponding emission lines are tabulated in Table 3. In summary, Al, Ca, and Mg were found in all six samples. Five samples, namely, MPS2, MPS3, MPS4, MPS5, and MPS6, showed the presence of Co with characteristic emission lines. Emission lines corresponding to Ni were found in five samples, namely, MPS1, MPS2, MPS3, MPS5, and MPS6. Zn was found in MPS1, MPS2, MPS4, MPS5, MPS6.

**Table 3:** Elements detected by LIBS analysis (excluding C, O, and N).

Trace element	Sample	Emission line (nm)
Ca	MPS 1–6	373.68, 393.41, 428.45
Mg	MPS 1–6	333.62, 383.81, 389.44
Al	MPS 1–6	394.39, 396.19
Co	MPS 2–6	343.3, 347.43
Ni	MPS 1–3, 5–6	323.34, 336.92, 356.65
Zn	MPS 1–2, 4–6	330.25, 334.49

#### EDS analysis of microplastics

**Figure 6:**
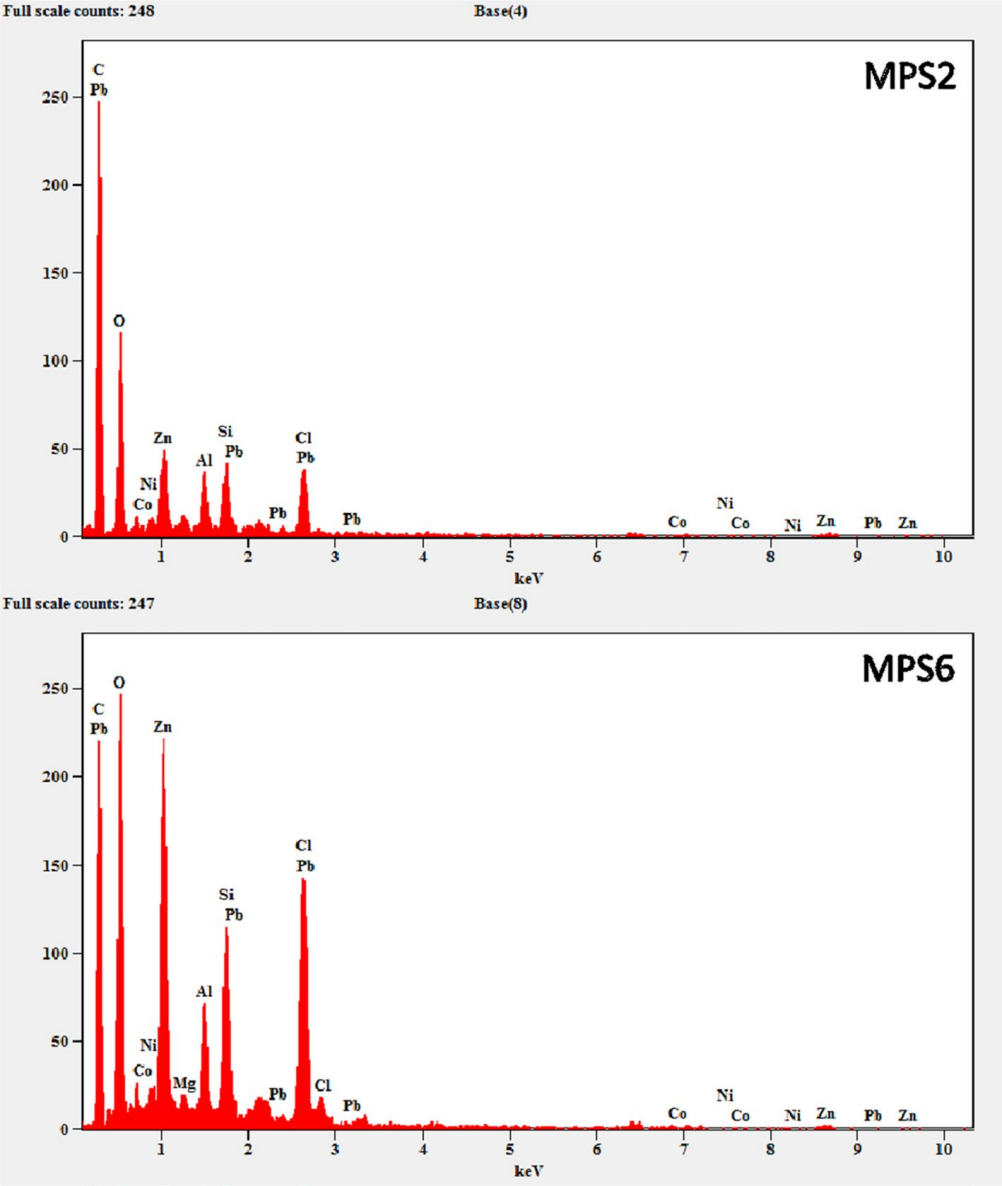
EDS spectra of two microplastics (MPS2 and MPS6) showing the presence of different surface adsorbed elements including the heavy metals.

Energy-dispersive X-ray spectroscopy (EDS) was employed using SEM coupled with an EDS system to cross-validate the presence of the elements identified by the LIBS. The summary of the results is tabulated in Table 4. Two sample EDS spectra of sample MPS2 and MPS6 are shown in Fig. 6. Most of the elements found in the LIBS analysis were also found in the EDS analysis. The major difference found was the presence of Pb, Si, and Cl in all the samples, which was not found in the LIBS analysis and might be because of the system's limit of detection. Apart from the above, Al was only found in samples MPS2 and MPS6, Mg was found only in MPS6, and Ca was only found in MPS4.

**Table 4:** Elements detected by EDS analysis (excluding C and O).

Trace elements	Sample
Si	MPS1, MPS2, MPS6
Cl	MPS1, MPS2, MPS6
Co	MPS1–6
Ni	MPS1–6
Zn	MPS1–6
Al	MPS2
Pb	MPS1–4, 6
Ca	MPS4
Mg	MPS6

The results clearly indicate the advantage of the proposed LIBS-Raman system in completely characterizing microplastics based on chemical composition and surface adsorbed heavy metals, and proven to be accurate based on the comparison with the results of conventional gold standard methods. The existing reports on microplastic analysis discuss these aspects of characterization via completely different sample preparation and analytical approaches, whereas the current results do not require additional complexities in the process^52–56^.

**Figure 7:**
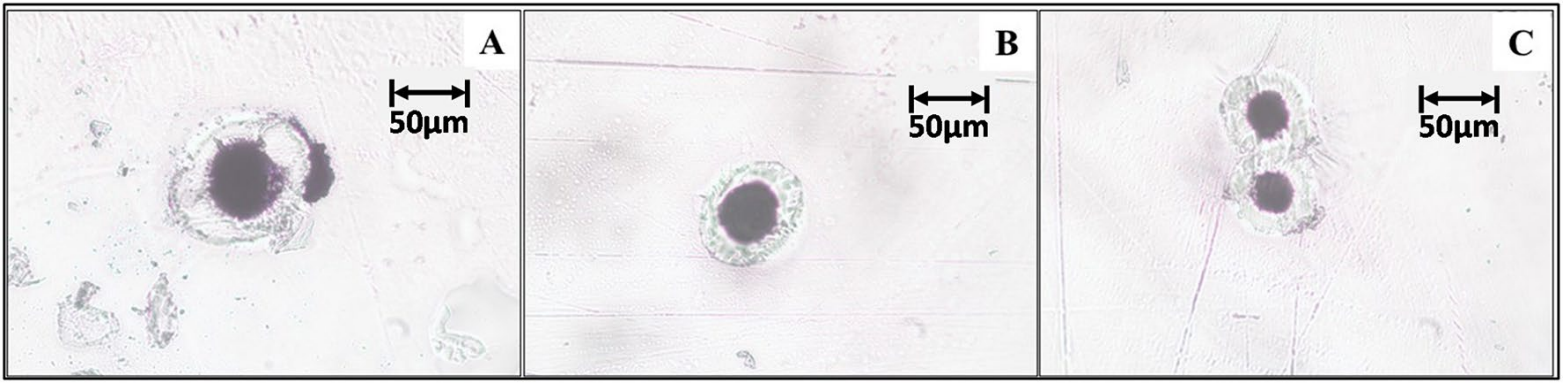
Craters created by the biconvex lens (**A**), 5× objective lens (**B**), and 10× objective lens (**C**).

An aspect of improving the reported system is incorporating a microscopic system in the configuration so that the characterization based on morphology can also be performed using the single system, and the analyzable size limit for the micro-particles can be brought further down. A comparison of ablation craters formed on the surface of a polymer material (polypropylene) when using the current focusing optics and microscope objectives of different magnifications are compared in Fig. 7, which clearly shows how the analyzable size can be brought down in the future.

## Conclusion

In summary, the microplastics collected from River Netravathi with multiple analytical systems have been characterized. Brightfield imaging and SEM characterization of microplastics were performed to characterize extracted microplastics based on the surface morphology. The roughed cracked surface with protrusions indicates the extinction of weathering. The microplastics with a size range of 1–5 mm were successfully characterized with the custom-built Raman and LIBS instrument, which reduces the utilization of multiple resources for the complete analysis of the sample. Out of six microplastics, two were identified as polyethylene (PE), two were identified as polypropylene (PP), and the remaining two were identified as polyethylene terephthalate (PET). The system's accuracy in identifying polymer class was cross-validated with confocal Raman spectroscopy and ATR-FTIR spectroscopy, and the results agree with characteristic bands of the corresponding polymer classes. LIBS analysis of microplastics detected the heavy metals, namely, Al, Ni, Co, and Zn, along with trace elements like Ca and Mg and EDS confirms these observations, in turn, proves the applicability of the developed technique for the identification of adsorbed heavy metals in microplastics. From the observed results, the system's ability to identify MPs and surface-adsorbed heavy metals significantly helps the microplastic research advance with rapid, complete characterization.

## Data Availability

The datasets used and/or analysed during the current study available from the corresponding author on reasonable request.
